# Hyperbaric area index calculated from ABPM elucidates the condition of CKD patients: the CKD-JAC study

**DOI:** 10.1007/s10157-014-0965-2

**Published:** 2014-03-30

**Authors:** Satoshi Iimuro, Enyu Imai, Tsuyoshi Watanabe, Kosaku Nitta, Tadao Akizawa, Seiichi Matsuo, Hirofumi Makino, Yasuo Ohashi, Akira Hishida

**Affiliations:** 1 Clinical Research Support Center, The University of Tokyo Hospital, Tokyo, Japan; 2 Nakayamadera Imai Clinic, Takarazuka, Hyogo Japan; 3 Third Department of Internal Medicine, Fukushima Medical University, Fukushima, Japan; 4 Department of Medicine, Kidney Center, Tokyo Women’s Medical University, Tokyo, Japan; 5 Department of Nephrology, Showa University School of Medicine, Tokyo, Japan; 6 Department of Nephrology, Nagoya University Graduate School of Medicine, Nagoya, Aichi Japan; 7 Department of Medicine and Clinical Science, Okayama University, Okayama, Japan; 8 Department of Biostatistics, School of Public Health, The University of Tokyo, Tokyo, Japan; 9 Yaizu City Hospital, Shizuoka, Japan

**Keywords:** Chronic kidney disease, Hypertension, Hyperbaric area index, Ambulatory blood pressure monitoring (ABPM), Blood pressure load

## Abstract

**Background:**

High prevalence of masked hypertension as well as persistent hypertension was observed in the Chronic Kidney Disease Japan Cohort (CKD-JAC) study. We proposed a novel indicator of blood pressure (BP) load, hyperbaric area index (HBI), calculated from ambulatory blood pressure monitoring (ABPM) data. The characteristic of this index and its relationship with kidney function were also evaluated.

**Methods:**

The CKD-JAC study, enrolled 2,977 patients, is a prospective observational study started in September 2007. ABPM was conducted in a sub-group from September 2007 to April 2010 and baseline ABPM data of 1,075 subjects (63.4 % male, 60.7 years old) were analyzed.

**Results:**

Mean systolic HBI of male and female patients were 242.3 and 176.5 mmHg×h, respectively. HBI sensitively reflected sex (54.7 mmHg×h higher in males than in females), seasonal effects (51.6 mmHg×h higher in winter than in summer), and advancing CKD stage [(16.5 mmHg×h higher) per −10 mL/min/1.73 m^2^ in eGFR]. The HBI was a significant factor to associate with reduced kidney function, after adjusting with nocturnal BP change (NBPC), sex, and other variables (*p* value <0.001).

**Conclusions:**

Our findings suggested that HBI might be a novel sensitive indicator for the reduction of kidney function, independent of patterns of NBPC.

## Introduction

Blood pressure (BP) is one of the most important risk factors for cardiovascular diseases (CVDs). The prevalence of chronic kidney disease (CKD) in Japanese adults has been estimated to be 13 % [1]. Patients with CKD are associated with high BP and, in turn, hypertension is an independent risk factor for developing of CKD [2, 3].

Ambulatory blood pressure monitoring (ABPM) has come to be used as a powerful medical examination device since late 80s, and various indicators calculated from ABPM data have been reported as novel predictive factors for several organ injuries [4–7]. The fluctuation in BP during a day, also known as nocturnal BP change (NBPC), has been focused and the relationship between NBPC and CVDs was studied. NBPC measurment indicates that it is insufficient for treatment of hypertension to achieve the optimal BP by using solely office BP. ABPM data can be used to evaluate diurnal variation. In addition, data can also produce a novel indicator to evaluate 24-h BP control from other viewpoints. One indicator is the hyperbaric area index (HBI). First reported in 1984 [8], it was believed to be an indicator of BP load. HBI is defined as the area encircled by polygonal line of ambulatory BP and the boundary line of hypertension. HBI did not judge hypertension from single BP measurement, but combined multiple BP measurements and time, based on BP variability [9]. However, HBI lacks clear definitions, such as, on which value the boundary line for hypertension is set up. In recent years, this index has been examined in a few studies targeting several diseases, such as hypertension [10] and diabetes complication [11]. In the past few years, attempts have been made to evaluate HBI as a predictive indicator in the field of pregnancy-induced hypertension [12, 13].

We recently reported the high prevalence of masked hypertension in CKD population and the association between NBPC and the reduction of kidney function using the ABPM data in the CKD Japan Cohort (CKD-JAC) study [14]. In this paper, our aim was to first clarify characteristics of HBI and its clinical importance using CKD-JAC data at the time of enrollment and then examine the correlation between HBI and NBPC, and their effect on kidney function.

## Materials and methods

### About the CKD-JAC study

The CKD-JAC study was started in September 2007 to investigate CKD patients in Japan. 2,977 subjects were enrolled and followed until December 2012. A detailed description of this study has been published [15]. In brief, the CKD-JAC study subjects were (1) Japanese, (2) aged 20–75 years, and (3) CKD stage 3–5. Major exclusion criteria were (1) patients with polycystic kidney disease, HIV infection, liver cirrhosis, or cancer; and (2) transplant recipients and patients who have previously received dialysis.

### ABPM and patient questionnaire

**Fig. 1:**
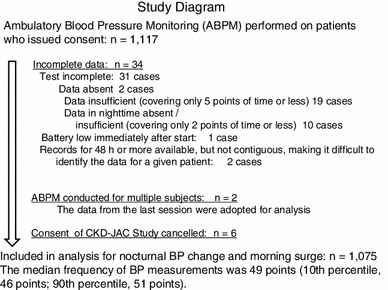
Target subjects. We had not set the exclusion criteria for ABPM. Protocol states the two following conditions: (1) patient consent was necessary for ABPM itself, separately from the consent to CKD-JAC enrollment. (2) Performed ABPM within half year from CKD-JAC study entry. According to the Japanese ABPM guideline, there was no set standard recommendation for how many time during the day or night to measure. Therefore, in our CKD-JAC, we manually examined all data from 1,117 patients and excluded the following 42 data from analysis

ABPM was conducted within a half year after starting observation. BP was measured every 30 min for 24-h period with the TM-2421 device (A&D Company, Japan). ABPM data were collected on 1,117 cases. Every case was visually inspected and 34 cases were determined to be invalid as examinations. Duplication was seen in 2 cases, and 6 subjects withdrew consent. Therefore, 1,075 cases were available for analyses (Fig. 1). A simple questionnaire was completed by each subject at the time of ABPM, and the questionnaire collected information such as the time to go to bed, the time to get up, the frequency of waking up to use lavatory, and the information about how the monitoring affected sleep.

Night time was defined as an actual sleeping time using subject’s diary. International Continence Society defined that nocturia as a individual condition to wake up one or more times at night to urinate [16]. In this study, when the subject woke up for urination three times or more during a night (20th higher percentile), the subject was defined to have “nocturia”. The sleep quality was rated on a 4-category scale from “as usual” to “much difficulty in sleep”.

The season for ABPM was divided into summer or winter according to data from the Chronological Scientific Tables by the National Astronomical Observatory of Japan. When the mean monthly temperature in the region of the participating facility was 20 °C or more, it was determined as in summer, and when it was less than 20 °C, in winter.

### Index calculated from ABPM

Following indexes were stratified from ABPM; NBPC, its patterns (extreme-dipper, dipper, non-dipper, and riser) and morning BP change. The degree of NBPC was calculated by the following equation:

$$\begin{aligned} {\text{The degree of NBPC}} & = 100 \, \times {\text{ [(mean daytime systolic pressure}}) - ({\text{mean nocturnal systolic pressure}})] \\ & \quad /({\text{mean daytime systolic pressure}}). \\ \end{aligned}$$

Details including cutoff points of NBPC patterns and NBPC definition were described in our previous paper [14].

### Hyperbaric area index is a novel indicator calculated from ABPM

**Fig. 2:**
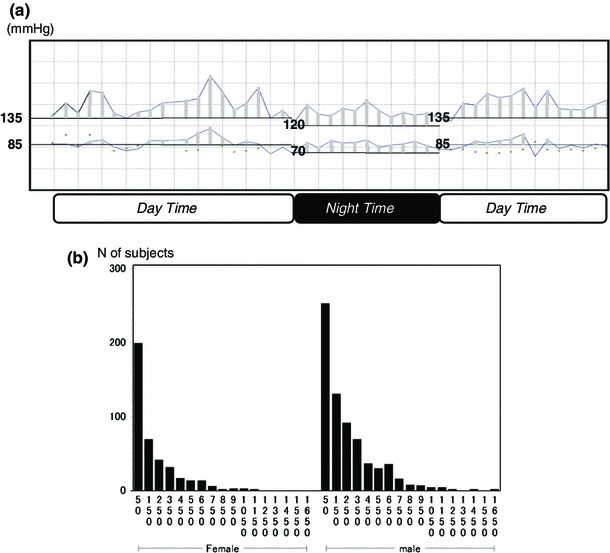
Hyperbaric area index (HBI). **a** Schematic representation of HBI. A trend graph was made from ABPM data (BP on *vertical axis* and time on *horizontal axis*) and the area of the graph [hyperbaric area (mmHg×h)] that exceeds baseline (135/85 mmHg when awaked and 120/70 mmHg when asleep) was calculated for systolic BP and diastolic BP. This value was adjusted per 24 h and used as HBI. **b** Distributions of HBI by sex. Distributions of HBI were right-skewed. However, HBI was analyzed with real number, because of more suited to clinical interpretation, after considering well the logarithmic transformation. Subjects were divided into two groups at the 75th percentile HBI value for each gender

Hyperbaric area (HB) was defined as the area encircled by polygonal line of ambulatory BP and two boundary lines of hypertension: 135/85 mmHg (during awakening) and 120/70 mmHg (during sleeping), based on Japanese Hypertension guidelines [17]. The area encircled by the ABPM trend graph and these two lines were defined as hyperbaric area (Fig. 2a). HB was calculated for systolic BP and diastolic BP, and HBI was defined as 24-h adjusted HB [18]. This was considered as an index of BP load on organs obtained from ABPM. As the HBI distribution was right-skewed, HBI above the 75th HBI percentile value for each gender was labeled as BP load (+) and HBI below that was labeled as BP load (−) for the sake of convenience. Since diastolic HBI was strongly affected by arteriosclerosis, we examined only systolic HBI for further analyses. It was analyzed with real number, without logarithmic transformation, for the sake of easy interpretation.

### Kidney function (eGFR and CKD stage)

Serum creatinine (Cre) from single blood sampling at the baseline was measured at a central laboratory and eGFR was calculated by the following Japanese equations [19]:

$${\text{Male: eGFR}}\,{\text{mL/min/1}}. 7 3\,{\text{m}}^{ 2} = 1 9 4 { } \times \left( {{\text{age}}^{ - 0. 2 8 7} } \right) \times \left( {{\text{serum Cre}}^{ - 1.0 9 4} } \right)$$

$${\text{Female: eGFR}}\,{\text{mL/min/1}}. 7 3 {\text{m}}^{ 2} = 0. 7 3 9\times 1 9 4\times \left( {{\text{age}}^{ - 0. 2 8 7} } \right) \times \left( {{\text{serum Cre}}^{ - 1.0 9 4} } \right).$$

CKD stage was defined using eGFR; 60 > eGFR ≥ 30 for stage 3, 30 > eGFR ≥ 15 for stage 4 and 15 > eGFR ≥ 10 for stage 5.

### Statistical analyses

All variables were reported as mean ± SD unless otherwise indicated. Continuous variables from two groups were compared with *t* test, and ANOVA was used for comparisons among more than 3 groups. For categorical variables, Chi-squared test (2 × 2 contingency table), or Cochran–Mantel–Haenszel test (*m* × *n* table) was performed. Simple linear regression analysis or Chi-squared test was used for univariate evaluations to investigate the relationship between ABPM parameters and background factors including patient questionnaires. Multiple regression analysis was used for multivariate evaluations including variables with *p* values <0.1 explored above. Two-way ANOVA was performed to investigate the relationship between kidney function and two indicators from ABPM (NBPC and HBI). The performance of SBP indicators as a discriminator for reduced kidney function was examined using receiver-operating characteristic curve (ROC) analysis.

All statistical analyses were performed using the SAS software program for Windows (version 9.2; SAS Institute Inc., Tokyo, Japan).

## Results

### Background

Table 1 summarized the subject’s characteristics. Of 1,075 subjects, there were 393 females (mean age 58.5) and 682 males (mean age 62.0). The mean BMI was 22.6 kg/m^2^ in female and 23.6 kg/m^2^ in male, and the mean office BP was 129.8/76.3 mmHg in female and 132.1/77.6 mmHg in male. The proportion of subjects according to CKD stage (female/male) was as follows: stage 3, 43.0 %/44.3 %; stage 4, 42.0 %/41.6 %; and stage 5, 15.0 %/14.1 %. Proteinuria was observed in 89.6 % of the female and 88.0 % of the male, and diabetes in 32.6 % of female and 37.1 % of male. Approximately 10 % of the subjects had not been prescribed even one antihypertensive drug.

**Table 1:** Characteristics of study participants

	Female	Male
Number of participants	393 (36.6)	682 (63.4)
Age (year)	58.5 ± 12.3	62.0 ± 10.6
CKD stage		
3	169 (43.0)	302 (44.3)
4	165 (42.0)	284 (41.6)
5	59 (15.0)	96 (14.1)
eGFR (mL/min/1.73 m^2^)	28.7 ± 12.6	28.8 ± 11.9
BMI (kg/m^2^)	22.6 ± 4.3	23.6 ± 3.3
Overweight (BMI ≥25)	78 (19.9)	182 (26.7)
Obesity (BMI ≥30)	23 (5.85)	29 (4.3)
Antihypertensive medicine use	343 (87.3)	632 (92.7)
Office SBP (mmHg)	129.8 ± 18.6	132.1 ± 17.8
Office DBP (mmHg)	76.3 ± 11.2	77.6 ± 11.5
Nocturnal BP change pattern		
Extreme dipper	40 (10.2)	65 (9.5)
Dipper	141 (35.9)	254 (37.2)
Non dipper	148 (37.7)	260 (38.1)
Riser	64 (16.3)	103 (15.1)
Morning BP surge (≥40 mmHg)	55 (14.0)	92 (13.5)
Morning BP surge (mmHg)	21.6 ± 16.6	23.5 ± 16.3
Diabetes mellitus^a^	128 (32.6)	253 (37.1)
Proteinuria^b^	345 (89.6)	581 (88.0)
Nocturia	50 (12.8)	154 (22.8)
Much difficulty in sleep	75 (19.1)	143 (21.2)
Examination period		
Summer	102 (26.0)	188 (27.6)
Winter	291 (74.1)	494 (72.4)

Data were *n* (%) or mean ± SD. The data of 1,075 participants who underwent ambulatory blood pressure monitoring were summarized

*BP* blood pressure, *CKD* chronic kidney disease, *eGFR* estimated GFR, *BMI* body mass index, *SBP* systolic BP, *DBP* diastolic BP

^a^Diabetes mellitus was diagnosed when at least one of the following criteria was met: diabetes mellitus described as an underlying disease or complication of CKD as reported by a physician, hemoglobin A1c of >6.5 % (National Glycohemoglobin Standardization Program), or concomitant use of antihyperglycemic drugs including insulin

^b^Proteinuria was identified when the urinary albumin/creatinine ratio from spot urine was ≥30 (mg/g creatinine)

### Patient questionnaires and NBPC patterns during ABPM implementation

In patient questionnaires, 12.8 % of females and 22.8 % of males had nocturia. In addition, ~20 % of subjects reported that it was extremely hard to sleep due to the ABPM.

The breakdown of the NBPC patterns (female/male) was as follows: extreme dipper 10.2 %/9.5 %, dipper 35.9 %/37.2 %, non-dipper 37.7 %/38.1 %, and riser 16.3 %/15.1 %. Approximately 27 % of subjects had their measurements taken during summer (Table 1).

### HBI

HBI distributions by sex were showed in Fig. 2b. Among female subjects, the mean (SD) systolic HBI was 176.5 (208.1) mmHg×h; the median HBI, 96.9 mmHg×h; and the 75th percentile value, 249.4 mmHg×h. Among male subjects, the mean (SD) systolic HBI was 242.3 (252.5) mmHg×h; the median HBI, 159.3 mmHg×h; and the 75th percentile value, 359.1 mmHg×h.

We evaluated the relationship between HBI and background factors stratified by sex (Table 2). HBI increased with advancing CKD stage in both females (*p* = 0.03) and males (*p* < 0.001). HBI increased by 26.0 mmHg×h in females and 39.7 mmHg×h in males for every 10 mL/min/1.73 m^2^ decreasing in eGFR. HBI was high in cases when office SBP/DBP were high (*p* < 0.001), and it was significantly higher in winter than in summer (females: *p* = 0.003, males: *p* = 0.01). On the other hand, there were no significant differences between with and without much difficulty in sleep in both sexes.

**Table 2:** Characteristics of systolic hyperbaric area index (HBI)

	*N*	Female	*p* value	*N*	Male	*p* value
	393	176.5 ± 208.1		682	242.4 ± 252.5	
Categorical variables						
Age						
20	7	133.5 ± 224.4	0.008	6	158.6 ± 102.1	0.09
30	36	110.7 ± 183.4		31	141.6 ± 177.9	
40	46	145.8 ± 230.0		46	211.7 ± 225.1	
50	90	140.6 ± 168.9		146	224.6 ± 234.2	
60	130	193.8 ± 211.9		266	252.5 ± 265.0	
70	84	236.7 ± 222.7		187	268.6 ± 264.2	
CKD stage						
3	169	147.3 ± 181.9	0.03	302	196.7 ± 219.5	<0.001
4	165	188.6 ± 222.1		284	261.7 ± 260.9	
5	59	226.2 ± 228.0		96	328.8 ± 293.8	
Overweight						
No	315	161.1 ± 205.9	0.003	500	222.9 ± 238.1	<0.001
Yes	78	238.7 ± 206.5		182	295.8 ± 282.4	
Obesity						
No	370	168.2 ± 205.9	0.002	653	241.2 ± 253.8	0.59
Yes	23	309.0 ± 201.9		29	267.3 ± 224.2	
Antihypertensive medicine use						
No	50	158.5 ± 207.2	0.51	50	146.7 ± 162.3	0.005
Yes	343	179.1 ± 208.4		632	249.9 ± 256.9	
Nocturnal BP change pattern						
Extreme dipper	40	146.0 ± 169.0	<0.001	65	180.5 ± 175.4	<0.001
Dipper	141	133.3 ± 157.5		254	197.0 ± 216.9	
Non dipper	148	172.1 ± 213.8		260	263.9 ± 254.8	
Riser	64	300.8 ± 263.2		103	338.7 ± 326.8	
Morning BP surge						
No	338	166.8 ± 205.3	0.02	590	235.2 ± 253.3	0.06
Yes	55	236.1 ± 217.2		92	288.5 ± 244.0	
Diabetes mellitus						
No	265	139.0 ± 187.9	<0.001	429	195.3 ± 213.6	<0.001
Yes	128	254.0 ± 226.6		253	322.2 ± 291.0	
Proteinuria						
No	40	66.5 ± 82.8	<0.001	79	126.2 ± 149.0	<0.001
Yes	345	190.0 ± 215.7		581	258.4 ± 257.9	
Nocturia						
No	341	163.9 ± 200.9	0.003	523	224.7 ± 246.7	<0.001
Yes	50	257.9 ± 238.1		154	302.1 ± 264.1	
Much difficulty in sleep						
No	317	169.4 ± 199.8	0.15	532	239.0 ± 150.6	0.71
Yes	75	208.3 ± 239.7		143	247.9 ± 255.1	
Season						
Summer	102	124.3 ± 160.0	0.003	188	201.8 ± 221.6	0.01
Winter	291	194.8 ± 219.8		494	257.8 ± 261.9	
Continuous variables						
Age (year)		30.3 (13.6, 46.8)	<0.001		29.0 (11.1, 46.8)	0.002
eGFR (10 mL/min/1.73 m^2^)		−26.0 (−42.2, −9.8)	0.002		−39.7 (−55.4, −24.0)	<0.001
SBP (10 mmHg)		52.6 (42.8, 62.4)	<0.001		58.5 (48.9, 68.2)	<0.001
DBP (10 mmHg)		45.8 (27.8, 63.7)	<0.001		39.2 (22.9, 55.6)	<0.001
24-h mean SBP (5 mmHg)		58.5 (55.8, 61.2)	<0.001		67.9 (65.6, 70.1)	<0.001
24-h mean SBP (10 mmHg)		117.0 (111.7, 122.4)	<0.001		135.7 (131.3, 140.1)	<0.001
BMI (1 kg/m^2^)		11.2 (6.6, 15.8)	<0.001		9.0 (3.1, 14.9)	0.003
Nocturnal BP change (10 %)		−60.9 (−83.1, −38.7)	<0.001		−61.1 (−82.2, −40.0)	<0.001
Morning surge (10 mmHg)		14.2 (1.7, 26.6)	0.03		5.5 (−6.2, 17.1)	0.36

Data were mean ± SD unless otherwise indicated. The relationship between HBI and individual factors was evaluated in males and females. The *p* values for continuous variables were used *t* test (two groups) or an analysis of variance (three or more groups), and the *p* values for categorical variables were used simple liner regression analysis

Sex and other ten variables with *p* value ≤0.1, including eGFR, proteinuria, and season, were taken into multiple regression model as independent variables so that we could assess their effects on HBI (Table 3). It should be noted that similar indicators were represented by a variable that was easy to interpret clinically. For example, kidney function was expressed by eGFR. HBI increased with eGFR decreasing (*p* = 0.003) and was 54.7 mmHg×h higher in males than in females. Subjects with proteinuria had higher mean HBI than subjects without proteinuria by 43.5 mmHg×h (*p* = 0.05), and subjects whose measurements were taken in the winter had higher mean HBI than subjects whose measurements were taken in summer by 51.6 mmHg×h (*p* < 0.001). ABPM examination itself interfered with the sleep of some subjects, but the relationship between sleep and HBI values was not significant (*p* = 0.71).

**Table 3:** Characteristic of systolic hyperbaric area index (HBI): multivariable analysis

	Difference in systolic HBI (mmHg×h)	*p* value
Male(versus female)	54.7	<0.001
Age (10 years)	2.4	0.70
eGFR (10 mL/min/1.73 m^2^)	−16.5	0.003
Proteinuria	43.5	0.05
Diabetes mellitus	72.6	<0.001
BMI (kg/m^2^)	5.8	0.001
SBP (10 mmHg)	44.0	<0.001
Nocturnal BP change (10 %)	−47.1	<0.001
Nocturia	46.4	0.007
Much difficulty in sleep	−5.8	0.71
Winter (versus Summer)	51.6	<0.001

Explanatory variables were chosen with sex and *p* value of ≤0.1 on univariate analysis. If there were several variables which were almost same index, a variable was chosen as the indicator on the basis of being easy to interpret clinically, such as eGFR as a representative for renal function, BMI for obesity and nocturnal BP change (NBPC) for journal BP fluctuation

### Difference among office SBP, 24-h mean SBP, and HBI for detecting reduced kidney function

**Fig. 3:**
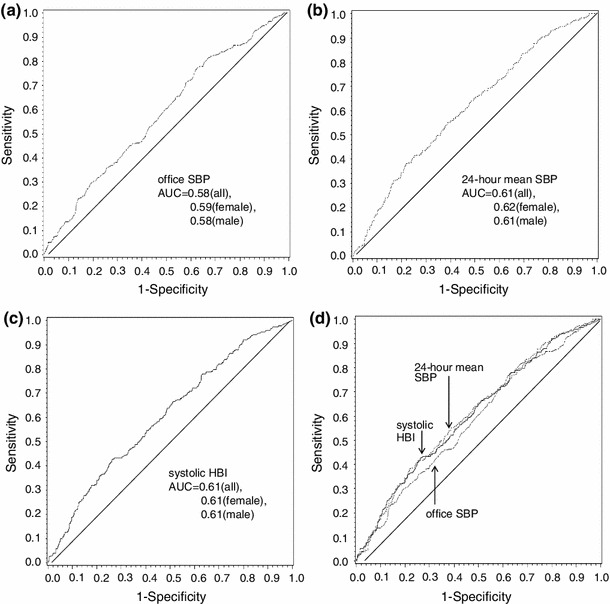
ROC curve analysis to discriminate low renal function ROC curves for office SBP (**a**), 24-h SBP (**b**), HBI (**c**) and all of them (**d**). Decreased renal function was defined as 25th eGFR percentile or lower. AUCs of office SBP were 0.58/0.59/0.58 (all/female/male), those of 24-h SBP were 0.61/0.62/0.61 (same as above) and those of systolic HBI were 0.61/0.61/0.61 (same as above). Since there are not apparent differences among ROC curves of all subjects, females and males, only ROC curves of all subjects were shown. Nonparametric approach to compare these three ROC curves was performed and office SBP was used as the reference. *p* value between office SBP and 24-h mean SBP was 0.16/0.40/0.27 (all/females/males), and that between office SBP and HBI was 0.23/0.71/0.25 (same as above). (- — - office SBP; - - - - 24-h mean SBP; —— systolic HBI)

We evaluated the performance characteristics of three BP indicators on kidney function: office BP, 24-h mean BP, and HBI using ROC curve (Fig. 3). Reducing kidney function was defined as 25th eGFR percentile or lower. Figure 3a shows the ROC curve for office SBP, Fig. 3b for 24-h mean BP, and Fig. 3c for HBI. Areas under the curves were 0.58, 0.61, and 0.61 for each. *p* value between office SBP and 24-h mean SBP was 0.16, and that between office SBP and HBI was 0.23.

### The relationship between HBI, NBPC, and eGFR

**Fig. 4:**
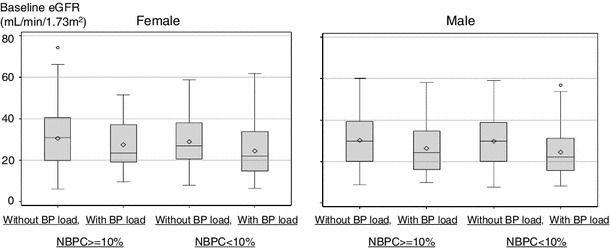
*Box*-and-*whisker* plots on eGFR for males and females. Subjects were divided into four groups by NBPC (<10 % or ≥10 %) and with/without BP load, and the *box*-and-*whisker* plots on eGFR were made to clarify the difference among them. The *length of the box* represents the interquartile range (the distance between the 25th and the 75th percentiles). The *dot in the box interior* represents the mean. The *horizontal line in the box interior* represented the median. The *vertical lines issuing from the box* extended to the minimum and maximum values of the analysis variable. If the minimum value was under *lower* fence or the maximum value is over *upper* fence, these observations were plotted. *Upper* fence is 1.5 interquartile range (IQR) above 75th percentile and *lower* fence was 1.5 IQR below 25th percentile

Finally, we examined the relationship between two ABPM indicators (HBI and NBPC) and eGFR at the same time point. First, patients were divided into two groups by NBPC: one is sufficient NBPC group with dipper or extreme-dipper, and the other is insufficient NBPC group with non-dipper or riser. And then each group is divided into two groups by with/without BP load (Fig. 4). eGFR was lower in subjects with high BP load than with low BP load, even if they had sufficient NBPC. The same tendency was observed with males and females, that is, the median eGFR is lower with BP load (+) than BP load (−) both in the group of sufficient NBPC (NBPC is 10 % or over) and in the group of insufficient NBPC, and median eGFR was the lowest in the group categorized with insufficient NBPC and with high BP load.

We then examined the relationship between NBPC or BP load and eGFR by two-way analysis of variance upon due consideration of the interaction between NBPC and BP load (Table 4). NBPC was not significantly associated with eGFR (females: *p* = 0.13, males: *p* = 0.37), whereas BP load was significantly associated with eGFR (females: *p* = 0.007, males: *p* ≤ 0.001). The interaction term between NBPC and BP load was not significant (females: *p* = 0.64, males: *p* = 0.58).

**Table 4:** Analysis of variance of the relation between eGFR and two indicators calculated from ambulatory blood pressure monitoring (ABPM)

Female	DF	SS	MS	*F* value	*p* value
Model	3	1872.7	624.2	4.03	0.008
Error	389	60242.6	154.9		
Corrected total	392	62115.3			

Female	DF	TypeII SS	MS	*F* value	*p* value
NBPC >10 %, <10 %	1	365.8	365.8	2.36	0.13
BP load <75 percentile, >75 percentile	1	1137.7	1137.7	7.35	0.007
Interaction term of NBPC and BP load	1	33.1	33.1	0.21	0.64

Male	DF	SS	MS	*F* value	*p* value
Model	3	3124.7	1041.6	7.57	<0.001
Error	678	93290.1	137.6		
Corrected Total	681	96414.8			

Male	DF	TypeII SS	MS	*F* value	*p* value
NBPC >10 %, <10 %	1	108.6	108.6	0.79	0.37
BP load <75 percentile, >75 percentile	1	2798.8	2798.8	20.34	<0.001
Interaction term of NBPC and	1	42.5	42.5	0.31	0.58

To determine the independent and combined effects of NBPC (<10 % or ≥10 %) and BP load (HBI <75 % percentile or ≥75 % percentile) on eGFR, two-way ANOVA was performed. The interaction terms of these two variables were not significant in either males or females

*DF* degrees of freedom, *SS* sum of squares, *MS* mean square

Next, we conducted multiple regression analysis including the continuous values of these two factors (the degree of NBPC: increments of 10 %, BP load: increments of HBI 100 mmHg×h) as well as sex and age as independent variables, and eGFR as a dependent variable (Table 5, left). 10 % decrease in NBPC corresponded to 0.48 mL/min/1.73 m^2^ decrease in eGFR (*p* = 0.08), while 100 mmHg×h increase in HBI corresponded to 0.72 mL/min/1.73 m^2^ decrease in eGFR (*p* ≤ 0.001). Another analysis using a model that included the season and the quality of sleep, both of which influenced the degree of NBPC, produced similar results (Table 5, right).

**Table 5:** Multiple regression analysis was performed with eGFR as a dependent variable

	Model A		Model B	
	Difference in eGFR (mL/min/1.73 m^2^)	*p* value	Difference in eGFR (mL/min/1.73 m^2^)	*p* value
Male (versus Female)	1.29	0.09	1.23	0.11
Age (10 years)	−2.15	<0.001	−2.13	<0.001
NBPC (10 %)	0.48	0.08	0.47	0.27
Systolic HBI (100 mmHg×h)	−0.72	<0.001	−0.70	<0.001
Much difficulty in sleep			−0.46	0.58
Winter (versus summer)			−0.73	0.41

Model A: sex, age, NBPC and BP load were included as independent variables. NBPC and HBI were dealt with as continuous values. The base unit was 10 % for NBPC, and 100 (mmHg×h) for HBI

Model B: Model A plus sleep quality and season

## Discussion

Recently, several studies showed that ABPM correlated more strongly with hypertensive target organ damage than office BP readings [20–22]. Yamamoto reported that CKD, ABPM, and small vessel diseases were independently associated with cognitive impairment in lacunar infarct patients [23].

In our previous paper, we reported that the prevalence of non-dipper or riser was lower among subjects with CKD stage 3 than in CKD stage 4 or 5. We also reported that when determining NBPC patterns, information regarding the season, the patient’s sleep quality, and nocturia should be taken into account [14]. After adjustment with these background factors, our study suggested that NBPC pattern might be an indicator of CKD prognosis. In this study, we have proposed HBI as another indicator for prognosis of CKD patients. On the basis of our results, we propose that HBI is a sensitive indicator of reducing renal function from ABPM data.

### Characters of HBI as an indicator of BP load

There was still insufficient solid evidence that HBI reflected the BP load on organs [24, 25]. Our data showed that HBI reflected sex, office BP, and kidney function extremely well, and it also reflected diabetes mellitus, proteinuria, and season. It suggested that HBI might be a quite sensitive indicator of BP load on kidney. As HBI was not found to be significantly affected by quality of sleep, it was unlikely that our HBI results were greatly modified by the stress of ABPM implementation. We found that HBI was largely affected by sex, with males having higher mean HBI values than females. This result was consistent with the fact that being male was a classical risk factor for CVD. Furthermore, what we wanted to emphasize is that HBI reflects the degrees of clinical findings as BP load and these findings can be compared quantitatively through the index.

### Two viewpoints, NBPC, and HBI, were needed when interpreting ABPM data

Subjects with non-dipper pattern of night-time blood pressure were reported to be associated with cardiovascular and cerebrovascular diseases [5, 26]. However, in this study, even in cases of sufficient NBPC, subjects with high HBI had reduced kidney function (Fig. 4). A similar trend was observed in subjects with insufficient NBPC. The group categorized with insufficient NBPC and with BP load had the lowest eGFR values.

In two-way analysis of variance (Table 4), the interaction term between NBPC and BP load was not significant (females: *p* = 0.64/males: *p* = 0.58). Hence, these two factors could be understood as having effects of BP on kidney function from different perspectives.

We also evaluated the relationship between these two factors and eGFR with multiple regression model adjusted with several background factors. As shown in Table 5, there was a strong correlation between HBI and eGFR (*p* < 0.001) and it was suggested that the effect of 100 mmHg×h HBI increase on eGFR was the equivalent of the effect of three-year increase in age on eGFR. 100 mmHg×h HBI increase was equivalent to mean BP increase of only 4.2 mmHg throughout 24 h. It could be realized that how effects of BP load on kidney function were great.

### Does HBI have superiority over office systolic BP in detecting reduced kidney function?

HBI has basically same meaning as office BP and 24-h mean BP. All of them are BP load to organs. We provided comparative performance measurements among them using ROC curves. ROC showed superiority of 24-h mean BP and HBI against office BP. Unfortunately, there were no significant differences between 24-h mean BP and HBI. However, these results indicated that it was insufficient to understand CKD patients’ BP control using solely office BP and that ABPMs were needed.

These results represented a first possible step towards evaluating BP load by HBI, because HBI strongly reflected background factors that may have association with kidney function. As next step, we will evaluate BP load by HBI accurately as a prognostic predictor for kidney function deterioration and CVDs by using prospectively collected data in the CKD-JAC study.

This paper was limited in that data analyzed were cross-sectional data at the enrollment. The last patient was out of this study in December 2012 and now we are carring out data cleaning.

## Conclusions

This study has clarified that HBI is able to separate the BP loads from background factors quantitatively. NBPC is one of the most useful indicators of the BP loads on clinical settings, and HBI may provide another index for this purpose. Because HBI was a sensitive indicator of kidney function, it also might be a predictor of future kidney function reduction, independent from patterns of NBPC. When the data cleaning has been completed, we will evaluate HBI as a prognostic indicator for kidney function and CVDs.
